# Involvement of PSMD10, CDK4, and Tumor Suppressors in Development of Intrahepatic Cholangiocarcinoma of Syrian Golden Hamsters Induced by *Clonorchis sinensis* and N-Nitrosodimethylamine

**DOI:** 10.1371/journal.pntd.0004008

**Published:** 2015-08-27

**Authors:** Md. Hafiz Uddin, Min-Ho Choi, Woo Ho Kim, Ja-June Jang, Sung-Tae Hong

**Affiliations:** 1 Department of Parasitology and Tropical Medicine, Institute of Endemic Diseases, Seoul National University College of Medicine, Seoul, Republic of Korea; 2 Department of Pathology, Seoul National University College of Medicine, Seoul, Republic of Korea; National Institute of Parasitic Diseases China CDC, CHINA

## Abstract

**Background:**

*Clonorchis sinensis* is a group-I bio-carcinogen for cholangiocarcinoma (CCA). Although the epidemiological evidence links clonorchiasis and CCA, the underlying molecular mechanism involved in this process is poorly understood. In the present study, we investigated expression of oncogenes and tumor suppressors, including *PSMD10*, *CDK4*, *p53* and *RB* in *C*. *sinensis* induced hamster CCA model.

**Methods:**

Different histochemical/immunohistochemical techniques were performed to detect CCA in 4 groups of hamsters: uninfected control (Ctrl.), infected with *C*. *sinensis* (Cs), ingested N-nitrosodimethylamine (NDMA), and both Cs infected and NDMA introduced (Cs+NDMA). The liver tissues from all groups were analyzed for gene/protein expressions by quantitative PCR (qPCR) and western blotting.

**Principal Findings:**

CCA was observed in all hamsters of Cs+NDMA group with well, moderate, and poorly differentiated types measured in 21.8% ± 1.5%, 13.3% ± 1.3%, and 10.8% ± 1.3% of total tissue section areas respectively. All CCA differentiations progressed in a time dependent manner, starting from the 8^th^ week of infection. CCA stroma was characterized with increased collagen type I, mucin, and proliferative cell nuclear antigen (PCNA). The qPCR analysis showed *PSMD10*, *CDK4* and *p16INK4* were over-expressed, whereas *p53* was under-expressed in the Cs+NDMA group. We observed no change in *RB1* at mRNA level but found significant down-regulation of RB protein. The apoptosis related genes, *BAX* and *caspase 9* were found downregulated in the CCA tissue. Gene/protein expressions were matched well with the pathological changes of different groups except the NDMA group. Though the hamsters in the NDMA group showed no marked pathological lesions, we observed over-expression of *Akt/PKB* and *p53* genes proposing molecular interplay in this group which might be related to the CCA initiation in this animal model.

**Conclusions/Significance:**

The present findings suggest that oncogenes, *PSMD10* and *CDK4*, and tumor suppressors, *p53* and RB, are involved in the carcinogenesis process of *C*. *sinensis* induced CCA in hamsters.

## Author Summary

*Clonorchis sinensis* is a helminth parasite and a carcinogenic agent for cholangiocarcinoma (CCA) or bile duct cancer in humans. Though a large and compelling body of evidence suggests an association between *C*. *sinensis* and CCA, the mechanism underlying at the genetic/proteomic level is little known. To explore the underlying molecular mechanism we investigated a number of genes/proteins in *C*. *sinensis* induced hamster CCA model. Here *C*. *sinensis* induced CCA successfully in all hamsters when introduced with N-nitrosodimethylamine. The histopathology confirmed the development of CCA and detected excessive collagen fibers, mucin and cell division related protein. The quantitative PCR analysis showed increased levels of oncogenes *PSMD10*, *CDK4* and decreased level of tumor suppressor gene *p53*. The western blot analysis observed significant decrease of another tumor suppressor called RB protein. Genes/protein expressions were matched well with the pathological changes of CCA hamster. The present study suggests that oncogenes, *PSMD10* and *CDK4*, and tumor suppressors gene *p53* and protein RB, are involved in the carcinogenesis process of *C*. *sinensis* induced CCA in hamsters.

## Introduction

The Chinese liver fluke, *Clonorchis sinensis* Looss 1907, is widely distributed in East Asia with some heavily endemic zones in China, Taiwan, Vietnam, Russia, and the Republic of Korea. In 2009, *C*. *sinensis* was reclassified as a group-I biocarcinogen for human cholangiocarcinoma (CCA) by the International Agency for Research on Cancer (IARC) based on epidemiological data [1,2]. Recently it was included in the control programs of neglected tropical diseases by WHO [3]. *C*. *sinensis* infection causes clonorchiasis, which is characterized by hyperplasia of biliary epithelium and metaplasia of mucin secreting cells in the intrahepatic bile duct [3]. The infection forms intrahepatic neoplastic lesion leading to mass forming CCA in Syrian golden hamsters when introduced with N-nitrosodimethylamine (NDMA) [4]. Syrian golden hamsters serve as a suitable model to study this parasite mediated carcinogenesis [4,5].

The molecular mechanism of *C*. *sinensis* induced CCA is little known [6] and it is crucial to understand its pathophysiology and to design efficient treatment strategy for CCA patients residing in the endemic region of the liver fluke. The development of CCA is a multi-step process at genetic level in which alterations of oncogenes and tumor suppressors as well as cell-cycle, apoptosis, and angiogenesis related genes are involved [7]. Recently it has been found that PSMD10 (also known as gankyrin), a regulatory subunit of 26S proteasome is upregulated in human CCA. It is also overexpressed in other types of cancer, including colorectal, pancreatic, and breast cancer. PSMD10 can regulate negatively most important tumor suppressors, p53 and retinoblastoma (RB). Its binding with CDK4 (cyclin-dependent kinase 4) further degrades RB.

The RB pathway is crucially important and found to be inactivated almost all types of human cancer [8]. *RB1*, *p16* ^*INK4*^ and *CDK4* are the major components of RB pathway, essential for cell cycle regulation specially for G1/S transition. *RB1* is the first tumor suppressor gene cloned in hereditary retinoblastoma. p16INK4 interferes the bindings with D-type cyclins as CDK4 or CDK6 inhibitor. Moreover, p16INK4 prompts p21 or p27 release from cyclin D-CDK complex. Differential expression of p16INK4 as well as CDK4 leads to dysregulated progression of cell cycle in many cancers. One study also demonstrated alterations of RB pathway related genes such as *RB1*, *p16* ^*INK4*^, *cyclin D1*, and *CDK4* in hamster CCA model of opisthorchiasis [9].

Beside these the apoptosis related genes were differentially expressed in the hamster CCA model [10]. Akt/PKB is the key driver of PI3K/Akt signal transduction pathway and its aberrant expression associated with malignancy [11]. Akt also inhibits apoptosis via the modulation of caspase 9. The apoptosis usually begins with BAX accumulation on the mitochondrial surface leading to the release of cytochrome c which binds to caspase 9 along with other molecules and initiates the cell death cascade [10]. It has been shown that suppression of *PSMD10* can cause apoptosis through BAX and caspase 9 mediated intrinsic pathway in hepatocellular carcinoma cells [12,13].

A comprehensive understanding of oncogenesis of *C*. *sinensis*-associated CCA is currently unknown, however, a previous study observed proliferative effect of *C*. *sinensis* excretory-secretory products (ESP) on different cell lines in vitro [14]. In this context, it is important to observe the changes of oncogenes and tumor suppressors at the molecular level. The present study investigated the involvement of oncogenes, *PSMD10*, *CDK4*; gene related to cellular proliferation, *Akt/PKB*, as well as tumor suppression, *p53* and *RB* in CCA induced by *C*. *sinensis* infection.

## Methods

### Ethics statement

The animal experiment protocol was reviewed and approved by the institutional animal care and use committee (IACUC) of Seoul National University, Seoul, Korea (SNU-100826-2) and followed the National Institutes of Health (NIH) guideline for the care and use of laboratory animals (NIH publication no. 85–23, 1985, revised 1996). It is accredited by the Ministry of Food and Drug Administration and also by the Ministry of Education, Science and Technology (LNL08-402) as an animal experiment facility. The laboratory has been monitored and inspected regularly by the Ministry and the IACUC of Seoul National University. Syrian golden hamsters, weighing about 70 g, were purchased from the Central Laboratory Animals Inc. (Seoul, Korea). Animals were housed at 21°C ± 2°C with 60% humidity and a 12-hour light-dark cycle, with free access to rat chow (Samtako Bio Korea Inc., Gyeonggi, Korea) and tap water. All of the experiments were conducted with an effort to minimize the number of animals used and the suffering caused by the procedures used in the present study. All manipulations of animals were carried out in animal biosafety level-2 (ABL-2) facilities in accordance with ABL-2 standard operating practices. Hamsters were euthanized with 2–3 times doses of anesthetics (mixture of xylazine [10 mg/kg; Bayer, Korea] and zoletil-50 [30 mg/kg, Virbac, France]) followed by cervical dislocation as a subsequent secondary measure.

### Collection of metacercariae

The metacercariae of *C*. *sinensis* were collected from naturally infected freshwater fish *Pseudorasbora parva* in Korea. We purchased the fish by licensed fishermen by the local district government, Bureau of Agriculture and Fishery of Gyeongsangnam-do, Korea. The fish flesh was digested in 0.5% pepsin solution with HCl and *C*. *sinensis* metacercariae were isolated under stereomicroscopic identification [15].

### Experimental design

Male Syrian golden hamsters (*Mesocricetus auratus*) of 4–5 weeks old were divided randomly into 4 groups including 15 animals each. Group I (Ctrl.) was uninfected control, Group II (Cs) received 30 metacercariae of *C*. *sinensis*, Group III (NDMA) drank NDMA mixed water, and Group IV (Cs+NDMA) received both metacercariae and NDMA water. The hamsters of the Group II and IV were infected with the metacercarae by intra-gastric intubation, and those of the Group III and IV received NDMA at a concentration of 12.5 ppm in drinking water for 8 weeks *ad libitum*. After 4, 8, 12 and 16 weeks, the hamsters were sacrificed and checked for the pathological and molecular changes.

### Histopathology

#### Hematoxylin and eosin (H&E) staining

A middle portion of the left, median, and right lobes of all livers were prepared for histopathological analysis after fixation in neutral buffered formalin (buffered 10% formaldehyde, pH = 7.2). Paraffin sections of 4 μm thickness were processed for routine hematoxylin and eosin (H&E) staining. CCA tissue areas of each histopathological slide were measured and expressed as percentages.

#### Masson’s trichrome staining

Masson’s trichrome staining was performed on randomly selected slides for the visualization of collagen fiber. Briefly, formalin fixed tissue section was refixed in Bouin’s solution for 1 hour at 56°C after deparaffinization and rehydration. Slides were stained with Weigert’s iron hematoxylin solution and Biebrich scarlet-acid fuchsin solution for about 10 minutes, followed by phosphomolybdic-phosphotungstic acid solution and aniline blue staining. After differentiation in 1% acetic acid slides were mounted through dehydration steps.

#### Sirius red staining

For better visualization of collagen fiber specially type I, randomly selected sections were subjected to Sirius red staining. In brief, rehydrated slides were stained for 8 minutes with hematoxylin and washed under running tap water for 10 minutes. Then Sirius red solution A was applied for 1 hour, followed by washing with 0.5% acidic water. Red stained fibers were counted as collagen fibers.

#### Alcian blue staining

Modified alcian blue staining was performed for detection of mucinous substance in the hamster tissue. After deparaffinization and hydration tissue sections were stained with alcian blue for 30 minutes. Sections were counter stained with H&E. The blue colored area with typical H&E background was considered as mucin.

#### Rapid mucin staining

Rapid mucin staining was performed using a rapid mucin staining kit (Polysicences Inc., USA) according to manufacturer’s instruction. In short, after deparaffinization tissue sections were immersed in descending grades of alcohol and in distilled water. Sections were then treated with complete Wieget’s iron hematoxylin solution for 1 minute followed by rinsing in running tap water. Counterstaining was done using green-1724 solution for 3 minutes. After a quick rinse in 1% glacial acidic acid, the slides were subjected to 0.5% basic fuchsin solution for 4 minutes. Mucin of stained tissues showed red to pink with a green cytoplasmic background.

### Proliferative cell nuclear antigen (PCNA), collagen I and IV immunohistochemistry

To detect the PCNA, collagen I and collagen IV immunohistochemisty procedures were carried out using a Histomouse MAX kit (Zymed Laboratories, Invitrogen Immunodetection, San Francisco, CA). The antigen was retrieved through incubation with citrate buffer (pH = 6) at 100°C in a water bath for about 1 hour. At primary antibody application step, PCNA monoclonal antibody (1:100; Clone IPO-38, Cat#10004805, Cayman Chemical, Ann Arbor, MI) or collagen I mouse monoclonal antibody (1:100; anti-collagen I antibody [COL-1], Cat# ab6308, Abcam, Seoul, Korea) or collagen IV rabbit polyclonal antibody (1:100; Anti-Collagen IV, Cat# ab6586, Abcam, Seoul, Korea) was used. The 3-amino-9-ethylcarbazole (AEC) was utilized for the production of red to pink color with horseradish peroxidase (HRP) conjugated secondary antibody.

### RNA extraction and cDNA preparation

A portion of the liver tissues from the hilar region of the livers of all hamsters were snap frozen in liquid nitrogen and stored in it for extraction of RNA. Total RNA was isolated using RNeasy plus mini kit (Cat# 74134, Qiagen, Hilden, Germany) according to the manufacturer’s instructions. Reverse transcription of 1–3 μg of total RNA was done by Maxim RT premix (Cat# 25081, Gyeonggi-do, Korea) cDNA synthesis kit. The cDNA was kept at -70°C until use.

### Analysis of gene expression by real-time PCR assay

The primer pairs for *PSMD10* was designed using primer BLAST of NCBI and for *CDK4*, *p16INK4*, *RB1*, *p53*, *Akt/PKB*, *BAX*, *caspase 9* and the housekeeping gene, glyceraldehydes-3-phosphate dehydrogenase (*GAPDH*) were chosen from published articles. Published sequences were matched with the sequences of the GenBank and summarized in Table 1. SYBR Green I (Applied Biosystems, Waltham, MA) DNA binding dye was used as fluorophore. After PCR amplification, melting curve analysis was performed to verify the PCR products. The standard curve was prepared for the determination of PCR efficiency. Before real-time PCR, all of the reactions were confirmed as single band in agarose gel electrophoresis by conventional PCR. Single band was also observed after real-time PCR as well (S1 Fig). Gene expressions were calculated using 2^-ΔΔCT^ (Livak) method.

**Table 1:** Oncogenesis related genes targeted primer pairs for real-time PCR.

Genes	Sequences
	Upper line: forward primer 5’-3’
	Bottom line: reverse primer 5’-3’
*PSMD10*	TGTCTAAGGTCTGCAACCTGGCCT
	AGCATGCCCAGTGTAATGCTGTTC
*Akt/PKB*	CCCTTCTACAACCAGGACCA
	ATACACATCCTGCCACACGA
*RB1*	CAGATGGTGTGTAATAGTGACCGA
	TTTTTCAGGGGCTTGGGAG
*p53*	AAGGCGATAGTTTGGCTCCT
	CTGGGGTCTTCCAGTGTGAT
*p16INK4*	GCAACACCCAAGTAGCCAGAC
	CGCCAGAGTTTCCAAGAAGCC
*CDK4*	CACCCTCGTGTTTGAGCATA
	GTTTTCTGGTTTCAGGTCTCGG
*BAX*	AGCTGCAGAGGATGATTGCT
	CTCTCGGAGGAAGTCCAGTG
*Caspase 9*	GATGCTGTCCCCTATCAGGA
	GGGACTGCAGGTCTTCAGAG
*GAPDH*	GACATCAAGAAGGTGGTGAAGCA
	CATCAAAGGTGGAAGAGTGGGA

### Western blot analysis

To determine the oncogenesis related protein levels in the hamster tissue, proteins were extracted according to the conventional method. Briefly, about 100 mg of the tissue was taken from each sample and grounded to a powdered preparation with liquid nitrogen. Needle homogenization was performed. The samples then underwent a process of 10 minutes homogenization and/or sonication in the presence of tissue protein extracting solution (lysis buffer containing 50 mM Tris-Cl (pH = 8.0), 150 mM NaCl, 0.1% SDS, 100 μg/mL PMSF, 2 μg/mL aprotinin, 2 μg/mL leupeptin, and 1% NP40). After cooling on ice for 30 minutes, and centrifugation at 14,000 *g* for 5 minutes at 4°C, the supernatants were collected.

Proteins were separated (40 μg/lane) by sodium dodecyl sulfate-polyacrylamide gel electrophoresis (8–12% SDS-PAGE). The concentration of proteins was determined by bicinchoninic acid (BCA) protein assay (Pierce, Thermo Scientific, Rockford, IL) using Nanodrop-1000 (Thermo Scientific, Wilmington, DE) according to manufacturer’s instruction. After electrophoresis, the proteins were electro-transferred to polyvinylidene fluoride (PVDF) membranes, blocked in 5% non-fat milk for 1 hour at room temperature (RT) and washed with PBST (PBS with 0.1% Tween), and probed with following primary antibodies: CDK4 (C-22: SC-260, Santa Cruz Biotechnology Inc. Santa Cruz, CA), Akt1 (C-20: SC-1618, Santa Cruz, CA), p53 (FL-393: SC-6243, Santa Cruz, CA), RB (C-15: SC-50, Santa Cruz, CA), and actin (I-19: SC-1616, Santa Cruz, CA). The membranes were then incubated with HRP-conjugated anti-rabbit (1:4,000 dilution; Cat# 81–6120, Zymax, Camarillo, CA) or anti-goat (1:2000 dilution; Cat# P-0449, Dako, Ely, UK) secondary antibody. Finally the blots were treated with enhanced chemiluminescence reagents (WEST-ZOL Plus Kit, iNtRON Biotechnology, Seongnam, Korea) and exposed to X-ray film. The images were obtained by the transmission scanner with the internal control of the actin protein levels and relative quantitative analysis was carried out based on the image band density ratio with ImageJ software of NIH, Bethesda, MD.

### Accession numbers/ID numbers for genes and proteins

GenBank accession numbers for each gene were as follows; Organism: *Mesocricetus auratus*; *PSMD10*, AF443797.1; *Akt/PKB*, M94355.1; *RB1*, GQ246228; *p53*, Y08900; *p16INK4*, AF292567; *CDK4*, GQ246229; *BAX*, AJ582075.1; *Caspase-9*, NM_015733; *GAPDH*, U10983. Protein accession numbers (NCBI) for each protein were as follows; Organism: *Mus musculus*; Actin, P68134; Akt1, P31750; RB, P13405; TP53 P02340; CDK4, P30285.

### Data analysis and statistics

Data obtained from the experiments were analyzed by Microsoft Excel (Ed. 2007, USA), GraphPad Prism 5 and SPSS-19 statistical software. Comparisons of results were performed using a Student’s t-test. *P* values < 0.05 were considered as significant.

## Results

### Detection of CCA through histopathological examination

**Fig 1:**
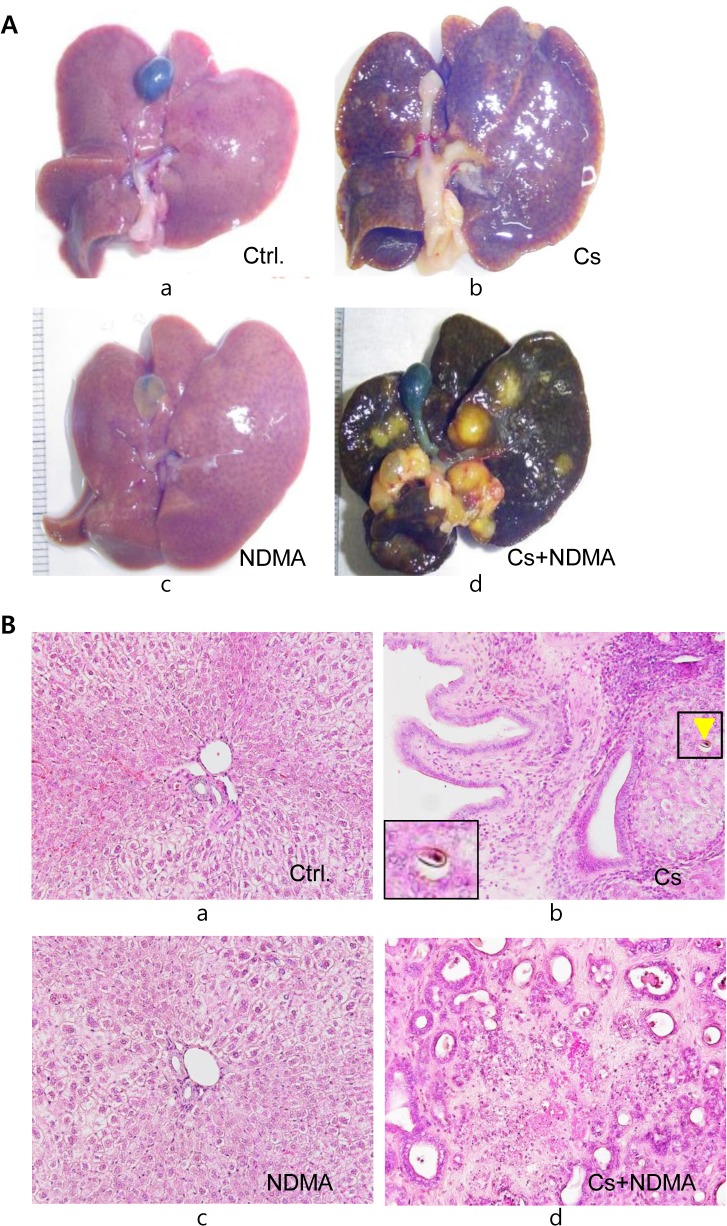
Gross view and histopathology of representative hamster liver by the groups. (A) Photographs of the liver after 16 weeks of infection (a-d represents Ctrl., Cs, NDMA, and Cs+NDMA group respectively). (B) H&E stained liver sections in different groups of hamsters (original magnification ×200) (a-d represents Ctrl., Cs, NDMA, and Cs+NDMA group respectively). Normal hepatic cells and biliary triad are observed in Ctrl. Group; proliferated bile duct, periductal fibrosis, and inflammatory cells are observed in the Cs group (yellow arrow head indicates an egg of *C*. *sinensis* enlarged below left); normal findings in NDMA group; highly proliferated bile duct epithelial cells in a fibrous stroma leading to CCA in Cs+NDMA group.

**Fig 2:**
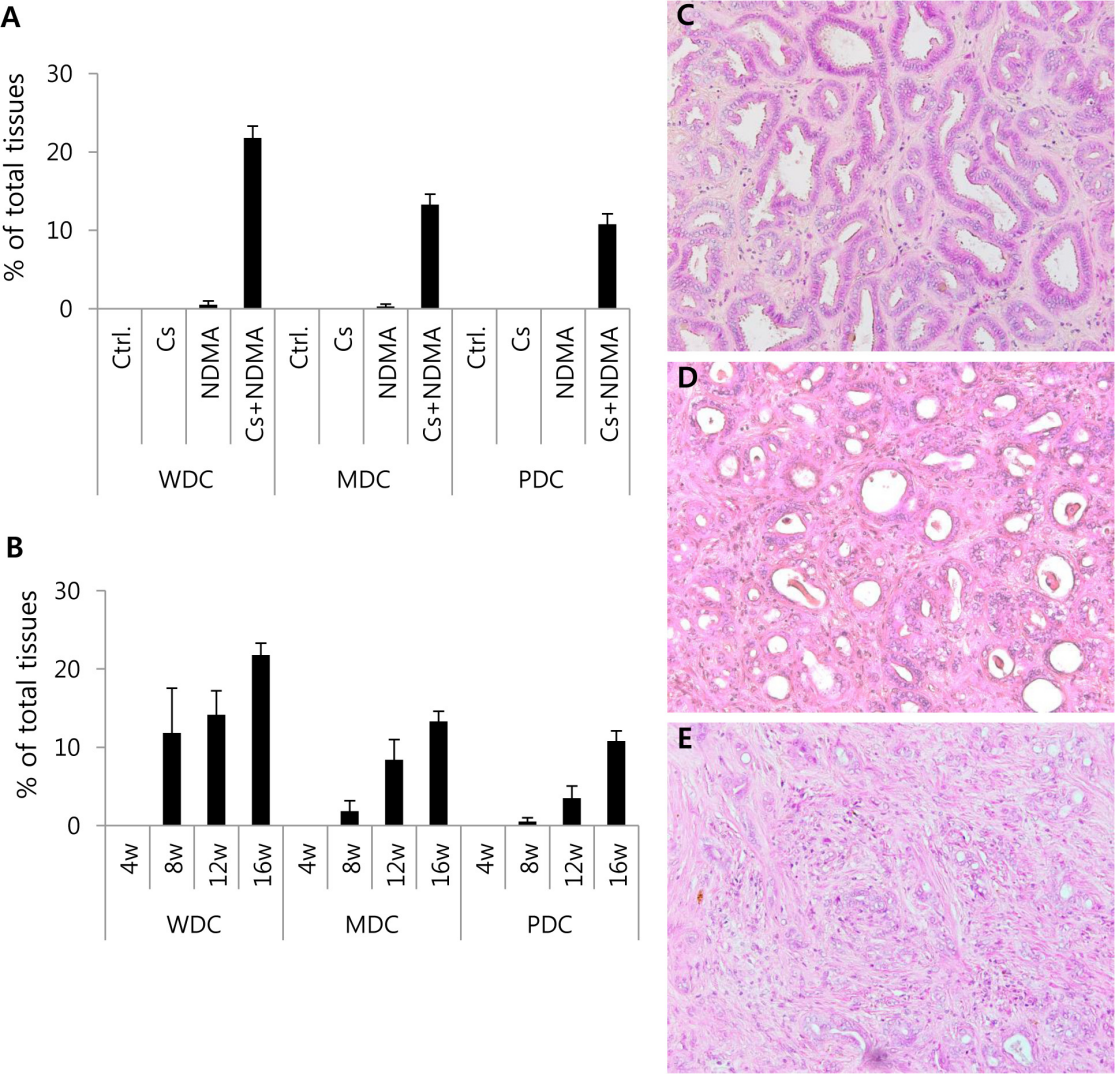
Semiquantitative neoplastic histopathological changes in different groups of hamsters. (A) Bar diagram showing well differentiated CCA (WDC), moderately differentiated CCA (MDC), and poorly differentiated CCA (PDC) in Cs+NDMA group. One hamster of NDMA group showed WDC and MDC in a tiny fraction of the liver. (B) Bar diagram showing progressive development of WDC, MDC, and PDC from 8^th^ weeks of infection. (C-E) Histopathological views of CCA (original magnification ×200): C, WDC; D, MDC; and E, PDC. Nuclear polymorphism of bile cells noted in C.

Mass forming lesions (MFL) were detected from gross observation of the liver’s surface and then from 2–4 mm slices of formalin-fixed livers. Representative slices from each lobe of the liver from each hamster were subjected to routine H&E staining (Fig 1). All hamsters in the Cs+NDMA group developed CCA leading to single or multiple MFLs. The CCAs originated in the Cs+NDMA group were categorized in well, moderately, and poorly differentiated types based on their histopathological features (Fig 2). In average, CCA was found in about 46% of the total tissue area of the Cs+NDMA group. Among CCA types, well differentiated CCA (WDC) was most prevalent and observed in 21.8% ± 1.5% of the total tissue area. Moderately differentiated CCA (MDC) was found in 13.3% ± 1.3% of the total tissue area and poorly differentiated CCA (PDC) was in 10.8% ± 1.3% of tissues (Fig 2A). Beside this, one hamster of the NDMA group showed WDC and MDC restricted in a very limited area (0.5% ± 0.5% and 0.03% ± 0.3% respectively) (S1 Table). We also observed a time dependent progression of CCA from the 8^th^ to 16^th^ week of infection in the Cs+NDMA group (Fig 2B). About 12% of the total tissue area was occupied by WDC at the onset of CCA at the 8^th^ week and increased to 14.2% and 21.8% after 12 and 16 weeks of infection respectively. A very tiny fraction showed PDC after 8 weeks of infection, however, it increased to 10.8% of the total tissue area after 16 weeks (S2 Table).

### Determination of characteristic CCA stroma

#### Collagen staining

**Fig 3:**
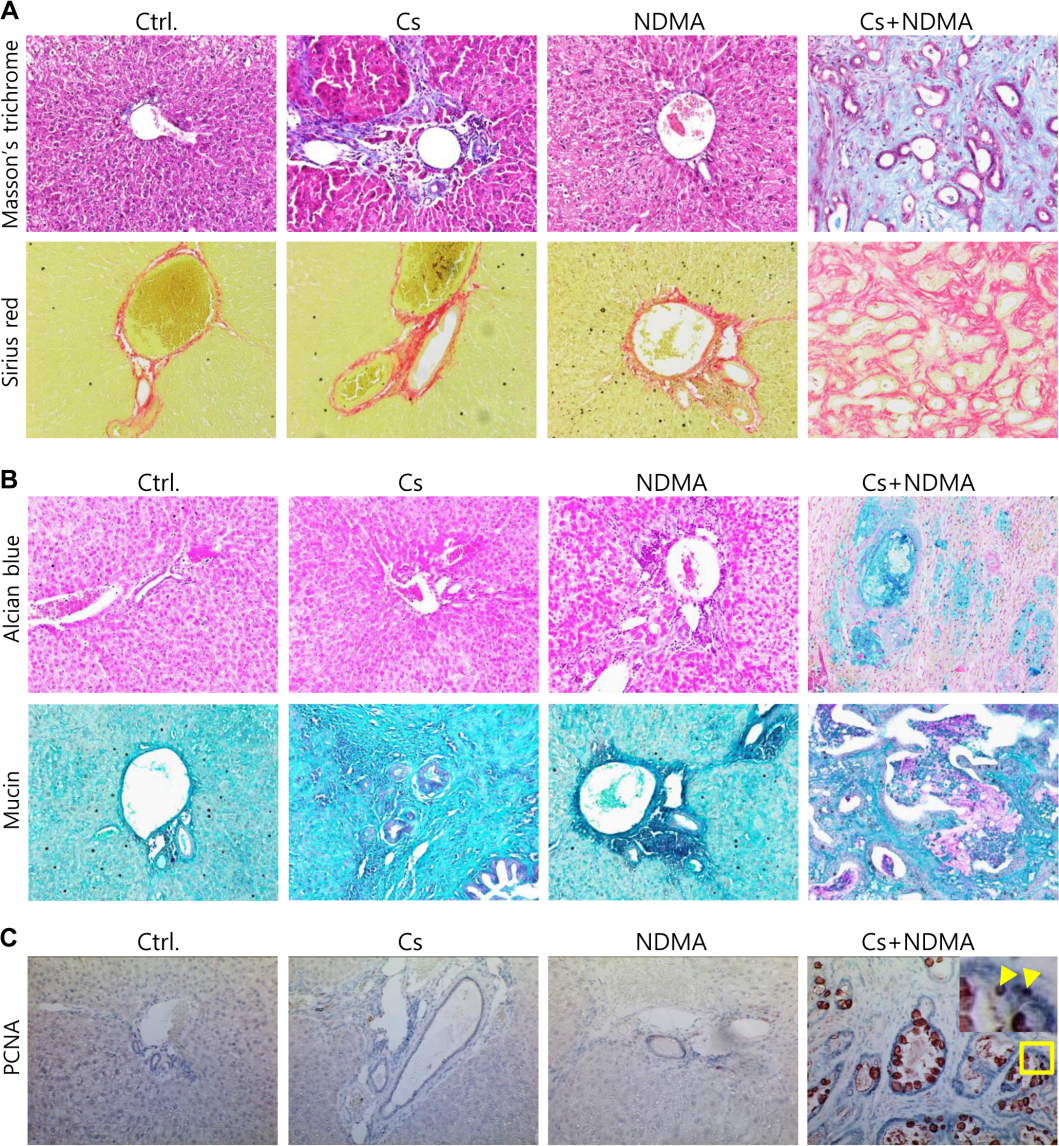
Collagen, mucin, and PCNA staining of liver tissue after 16 weeks of infection. (A) The livers of Ctrl. and NDMA groups show little collagen fibers (blue in Masson’s trichrome and red in Sirius red staining) at the portal triad. Hamsters in *C*. *sinensis* infected group (Cs) have collagen fibers around the bile duct, and dense collagen fibers are observed in Cs+NDMA group. (B) No stained cells or tissues are in the Ctrl. and NDMA group, but scanty or abundant mucin substances are detected in the Cs or Cs+NDMA groups (positive: blue by Alcian blue and red to pink in rapid mucin staining). (C) PCNA staining is strongly positive (red-brown) in Cs+NDMA group, demonstrating a higher number of proliferative cells (yellow arrowheads). Both large and small sized (enlarged) bile cells show positive reactions. Original magnification ×200.

Collagen depositing in CCA tissues is evident from a number of studies [16]. In the present study, the Masson’s trichrome staining recognized dense collagen fibers around the bile ducts in the Cs and Cs+NDMA groups. The least amount of collagen was found in the control and NDMA groups around the biliary triad. Sirius red staining showed very little collagen around the blood vessel and bile duct, periductal and bridging collagen in the Cs group and dense fibers around the bile ducts in the Cs+NDMA group forming fibrous stroma of CCA tissues (Fig 3A).

#### Mucin staining

Being a type of adenocarcinoma, CCA tissues produce varying quantity of mucin [17]. Both alcian blue and rapid mucin stainings were performed to see the presence of mucin in the liver sections (Fig 3B). Alcian blue showed characteristic slight blue depositions in the Cs group and extensive blue depositions in the Cs+NDMA group. In control and NDMA groups, no blue staining was observed. Rapid mucin staining also confirmed mucin in the Cs+NDMA group with huge pink areas in the tissue section (Fig 3B).

### Immunohistochemical confirmation of CCA by proliferative cell nuclear antigen staining

Proliferating cell nuclear antigen (PCNA) is a homotrimeric molecule, facilitates DNA polymerase δ and is essential for DNA replication [18]. It serves as a proliferative marker for different types of cancer and important in the context of genotoxic stress [19]. In the current study, immunohistochemistry showed strong positive reaction against PCNA antibody only in the Cs+NDMA group among 4 groups of hamsters. PCNA was mostly accumulated in the cells of bile duct epithelium and the stroma, which confirmed the development of CCA (Fig 3C).

### Immunohistochemical confirmation of collagen I and IV

**Fig 4:**
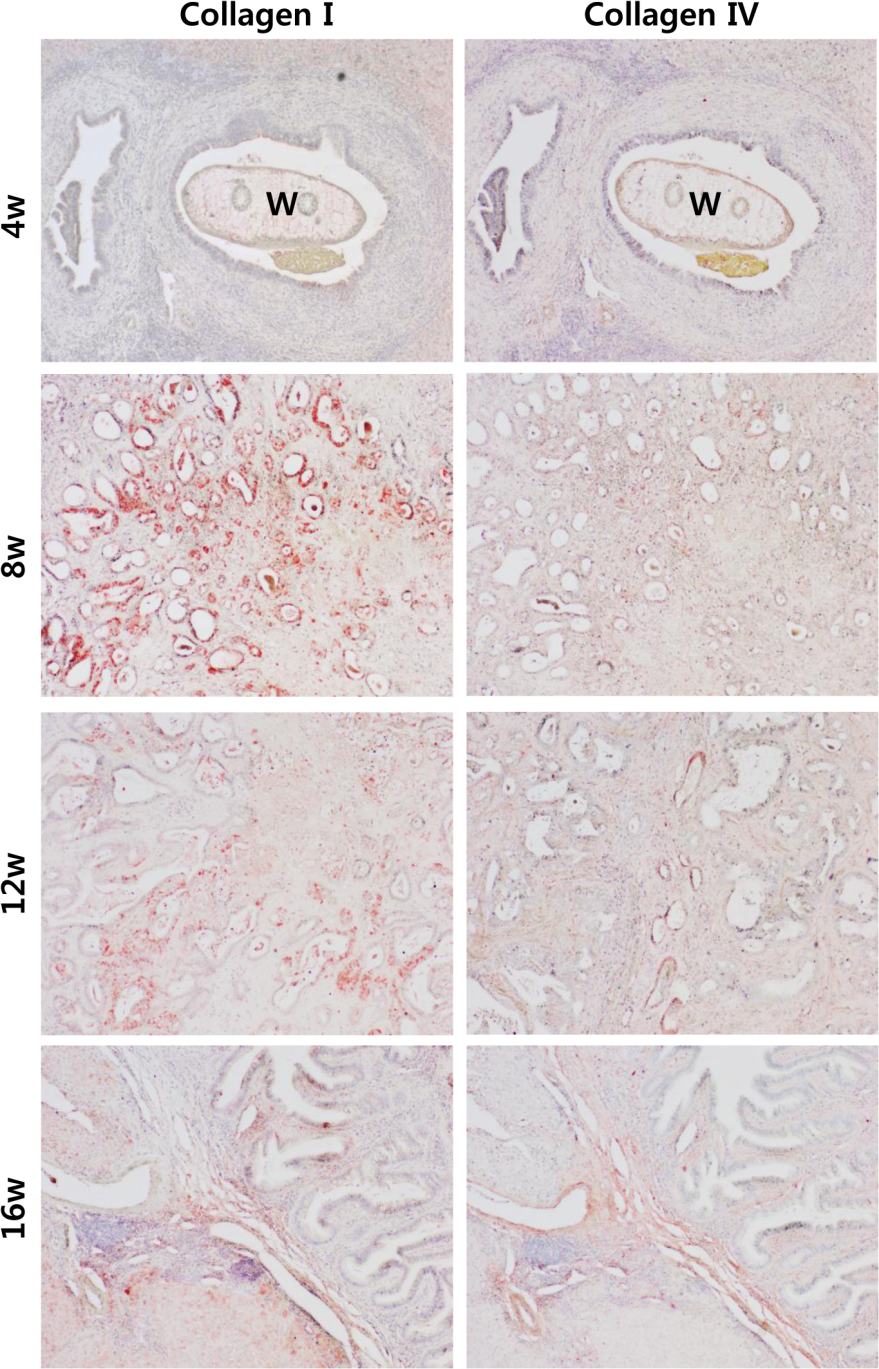
Immunohistochemistry of collagen type I and IV in Cs+NDMA group by infection duration. Collagen type I is deposited from 8^th^ to 16^th^ week and type IV at the 16^th^ week of infection (positive: red color). W = *C*. *sinensis* worm. Original magnification ×100.

The presence of collagen fibers type I and IV was evaluated using immunohistochemistry in the Cs+NDMA group. Though positive staining was not observed in the 4^th^ week of infection for collagen I but from the 8^th^ to 16^th^ week it showed strong positive staining. In the present study, we also observed positive reaction for collagen IV after 16 weeks of infection (Fig 4).

### Expressions of oncogenesis related genes/proteins

**Fig 5:**
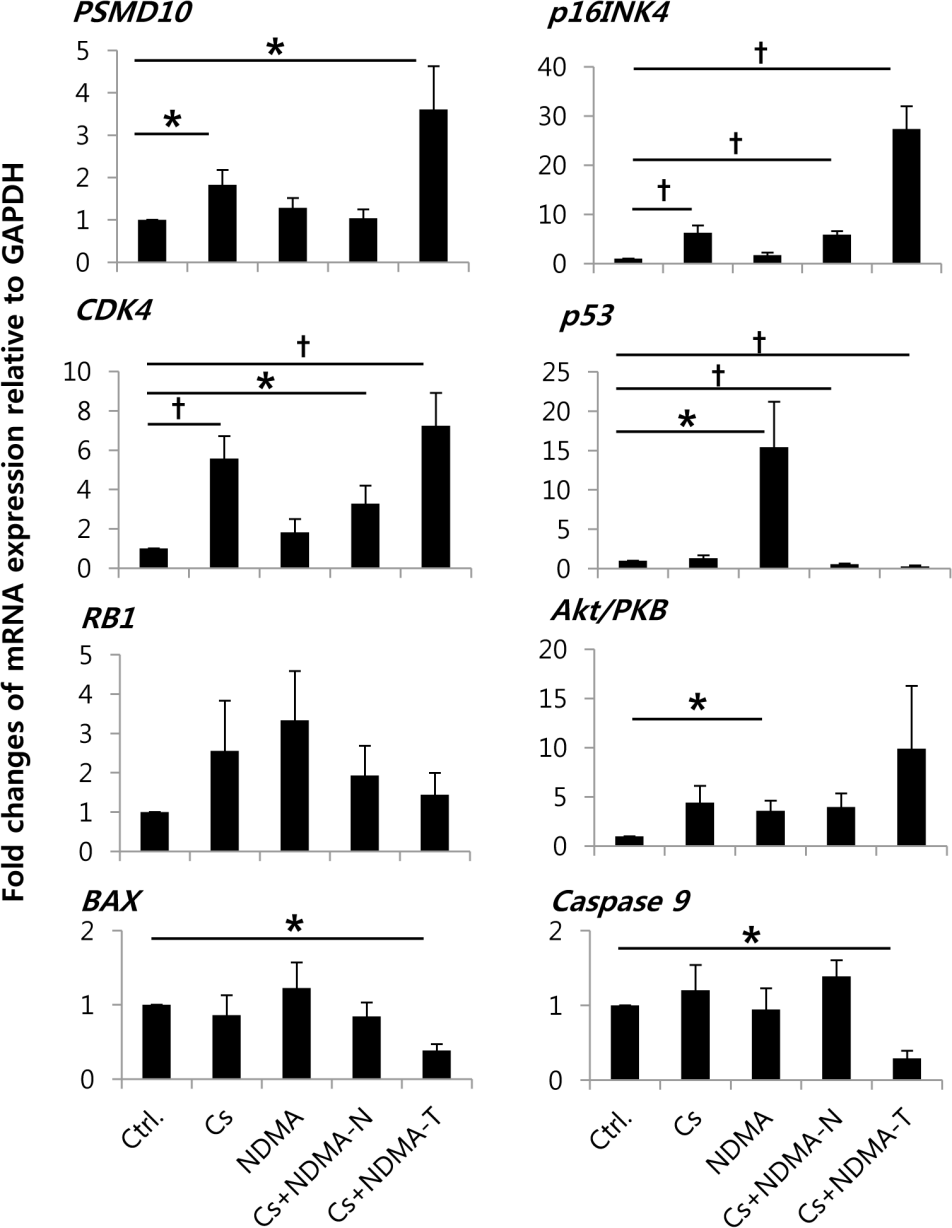
Relative mRNA expression of oncogenesis related genes by real-time PCR. The significant increase in the expression of *PSMD10*, *CDK4*, *p16INK4*, and decrease of *p53*, *BAX*, *caspase 9* genes are observed in the CCA tumor tissues (Cs+NDMA-T). *P* < 0.05 was considered as statistically significant. *P* < 0.05 and *P* < 0.001 indicated as asterisk and dagger respectively.

**Fig 6:**
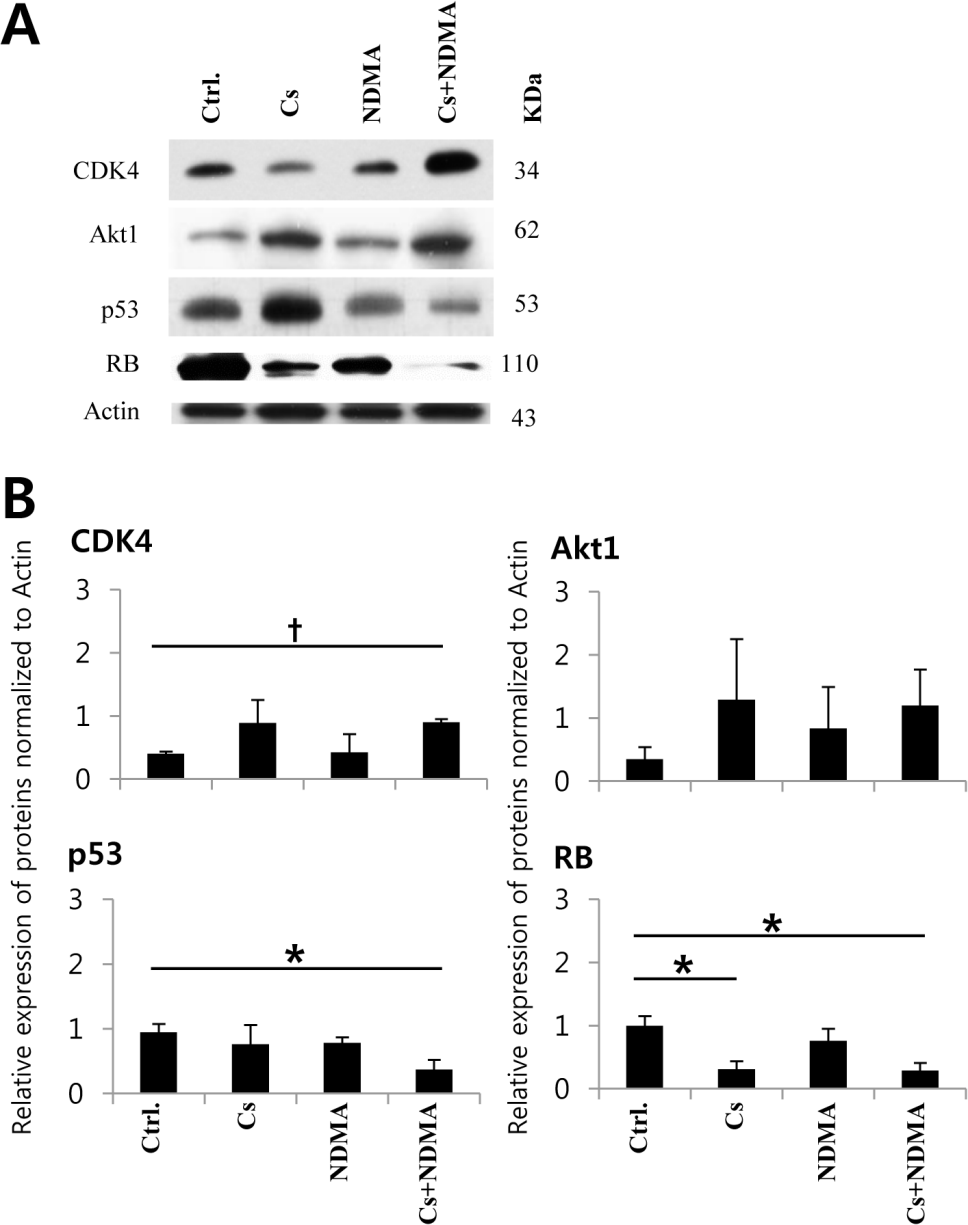
Relative expression of oncogenesis related proteins by western blot analysis by NIH—ImageJ software. (A) Protein bands representative of three independent experiments. (B) Increased expression of oncogene CDK4 in Cs and Cs+NDMA groups, and decreased expression of p53 and RB proteins in Cs+NDMA group are shown. *P* < 0.05 was considered as statistically significant. *P* < 0.05 and *P* < 0.001 indicated as asterisk and dagger respectively.

Differential expression of genes/proteins was recognized in CCA by a number of studies [10,20–23]. In the present study, the relative expression of mRNA by real-time PCR (Fig 5) showed that *PSMD10* and *CDK4* genes were over-expressed (*P* = 0.034; *P* = 0.006) in the tumor tissues of the Cs+NDMA group hamster’s liver. Tumor suppressor gene *p53* was downregulated both in the tumor (Cs+NDMA-T; *P* < 0.001) and adjacent normal tissues (Cs+NDMA-N; *P* < 0.001) but upregulated in the NDMA group (*P* = 0.036), and remained same in the Cs group. However, the other tumor suppressor *RB1* demonstrated no change in tumor tissue of the Cs+NDMA group (*P* = 0.440). *CDK4* inhibitor *p16INK4* showed upregulation in the tumor (*P* < 0.001) and adjacent normal tissues (*P* < 0.001) as well as in the Cs group (*P* = 0.007) but remained same in the NDMA group. An increase of *p16INK4* in Cs+NDMA group was almost 27 fold which was highly significant. *Akt/PKB* showed slight upregulation but it was not significant (Fig 5) (S3 Table). Apoptosis related genes, *BAX* and *caspase 9*, showed significant downregulation in CCA tissue (*P* = 0.002; *P* = 0.002) compared to that in the control. Western blot analysis using Akt1, CDK4, p53, and RB primary antibodies detected the proteins in the hamster tissues (Fig 6). Both p53 and RB proteins were found under-expressed in the Cs+NDMA group, however, CDK4 and Akt1 were increased. The increase of CDK4 (*P* = 0.001) and decrease of p53 (*P* = 0.043) and RB (*P* = 0.021) were statistically significant (Fig 6) (S4 Table). Significant downregulation of RB protein also observed in the Cs group (*P* = 0.024).

## Discussion

In the present study, we detected CCA masses in all hamsters of the Cs+NDMA group. The histopathological analysis revealed well, moderate, and poorly differentiated CCA with necrotic center. The CCA tissues contained abundant collagen type I and mucin, and showed PCNA overexpression. A multifunctional gene *PSMD10* and cell cycle regulatory oncogene *CDK4* along with tumor suppressors (*p53*, RB) were found to be expressed differentially in the CCA tissues suggesting their involvement in the process of CCA development in the *C*. *sinensis* and NDMA induced CCA hamster model.

MFL is the main pathological finding of *C*. *sinensis* and NDMA induced CCA in the hamster model. All of the hamsters of the Cs+NDMA group developed the MFL of CCA, which is the first report that observed 100% prevalence of *C*. *sinensis* induced CCA. Further analysis of MFL revealed that all of three grades of CCA (WDC, MDC and PDC) were mixed present at the later time of infection. The histologic progression of CCA has occurred in a sequence from WDC to PDC (Fig 2B) in a time dependent manner, starting from the 8^th^ week of infection. Such findings suggest that the CCA in this model begins from well differentiated type and later replaced gradually by poorly differentiated type. In the present study, we observed WDC and MDC in one MFL of the NDMA group (0.57% of total tissue area). Though the average areas were negligible, it suggested that CCA might be developed in the NDMA group when the 12.5 ppm dose of NDMA was introduced for 8 weeks and the hamsters were kept for 16 weeks. Optimization of dose and time for NDMA should be considered for the further improvement of this model.

Collagens are the major component of the stroma which normally maintains tissue integrity. Type I collagen was found to be associated with the progression of CCA in the animal model and considered as a predictive marker for *Opisthorchis viverrini* induced CCA in human [24]. The collagen fibers were also evident from squamous cell carcinoma, colon carcinoma [25,26] and serve as a distinguishing characteristic of hepatocellular carcinoma and CCA [27]. The collagen type I in extracellular matrix (ECM) interacts with certain molecules such as periostin and activates Akt signaling pathway in CCA [28]. In the present study, the presence of abundant collagen fibers in and around the CCA stroma suggests strong ECM interactions. Among collagens, collagen type I serves as binding site for type I transmembrane receptor tyrosine kinases within the ECM and helps to mediate different stromal/cellular signaling [29]. Strong immunohistochemical staining for type I collagen just after 8 weeks of infection indicates activation of a number of signaling pathways in development of CCA. The increased level of such fibrillary collagen was maintained throughout the study period. Besides collagen I, we observed collagen type IV overexpression lately at the 16^th^ week of infection which may indicate tumor invasion. Tumor cell survival, proliferation, and migration are highly dependent on ECM and collagen type IV which may serve as permissive substrates for tumor cell migration [30]. In gastrointestinal adenocarcinoma, elevated collagen IV level in peritoneal sample significantly associated with poor prognosis [31]. Therefore the presence of type IV collagen in the stroma of CCA might be due to the advancement of malignant CCA. The chronic inflammation caused by *C*. *sinensis* along with NDMA triggers activation of hepatic stellate cells, which may be precursors of both hepatocytes and cholangiocytes [30]. The inflammation and cellular activation can foster the collagen deposit.

The CCA is a type of adenocarcinoma [17] which produces mucin. We checked mucin production and observed ample amount of mucin in the CCA tissues both by alcian blue and rapid mucin staining. At the cellular level, we observed extensive conversion of cholangiocytes to mucin secreting cells in the Cs+NDMA group about 7 and 5 folds more than that of the Cs and NDMA groups respectively (*P* < 0.001; *P* = 0.001) (S2 Fig) (S5 Table). Such mucin production is evident from different types of adenocarcinoma involving the pancreas, lungs, breast, ovary, colon and other organs [32]. The mucin secretion is well-known in the hyperplastic biliary mucosa of clonorchiasis [3]. Basically mucin secretion is one of local protective reactions to the parasite in the mucosa. Higher numbers of mucin producing cells in the CCA tissue suggest that the mucin was continuously produced in the neoplastic tissue.

PCNA is an important marker for the detection of rapidly dividing cells in many cancerous tissues [33]. Our observation of strong positive reactions against PCNA in Cs+NDMA group but not in the tissues of other groups indicates higher proliferation activity in that particular group. PCNA can be a marker molecule of CCA in human clonorchiasis, which requires further evaluation.

The present study recognized overexpression of a novel oncogene named *PSMD10* in *C*. *sinensis* mediated CCA. Expression of *PSMD10* was shown in endometrial, breast, and colorectal cancer [34–36] and very recently in human CCA [37]. Infection with *C*. *sinensis* can be one of the reasons for such increase. *p16INK4* was downregulated in earlier studies with *O*. *viverrini* [9], but we observed a 6-fold increase in the Cs and 27-fold in the Cs+NDMA group. Overexpression of *p16INK4* was evident from cervical intraepithelial neoplasia [38]. The overexpressed *p16INK4* failed to suppress CDK4 which indicated possible aberrant changes in this gene. In western blot analysis, the expression of RB protein was significantly decreased, which suggests a possible mutation in *RB1* or rapid degradation of RB protein. Such mutated RB pathway gene can cause overexpression of *p16INK4* [39,40]. Moreover, a study also suggested association of *PSMD10* with the degradation of RB protein [41]. The *p53* plays an important role in both tumor suppression and apoptosis. Previous studies found the overexpression of *p53* in early infection of *O*. *viverrini* [10,42], however, after long term infection with *C*. *sinensis* and NDMA treatment, *p53* was downregulated in our model. In addition, *CDK4* was upregulated significantly. Upregulation of oncogenes *PSMD10* and *CDK4* and downregulation of tumor suppressor *p53* and RB confirmed carcinogenic changes at the genetic level in the present CCA model. One of the important hallmarks of cancer is avoidance of apoptosis. *BAX* localization on the mitochondrial membrane causes the release of cytochrome c release, which in turn activates the caspase cascade resulting apoptotic cell death [12]. In the present study, downregulation of apoptosis related genes, *BAX* and *caspase 9* has occurred probably by the upregulation of *PSMD10* [13]. Further study should be performed to elucidate the signaling pathway involving genes/proteins PSMD10, CDK4, p53, and RB. The possible interactions between the genes/proteins in the development of CCA are summarized in S3 Fig.

In conclusion, the underlying mechanism of CCA development relies on the alteration at the genetic level, which varies depending on the etiological agents. Continuous mechanical and chemical irritation by *C*. *sinensis* and NDMA may cause genetic alterations. Such accumulated genetic changes produce aberrant proteins leading to neoplastic transformation. An upregulation of *PSMD10* and *CDK4* genes along with the downregulation of tumor suppressor gene *p53* and protein RB is more likely to be associated in *C*. *sinensis* and NDMA induced transformation of bile duct epithelial cells in Syrian golden hamsters. Downregulated *BAX* and *caspase 9* may make more survival of the transformed cells possible, and the transformed cells initiate uncontrolled proliferation to form CCA mass. Overexpressed PCNA is detectable from the tumor tissue, which may serve as a marker of CCA in clonorchiasis.

## Supporting Information

S1 Fig Real-time PCR using SYBR Green I DNA binding dye. (A) Real-time amplification curve with negative RT product. (B) Melting peak chart of same amplified sample. (C) Agarose gel electrophoresis of same PCR product for the confirmation of single band (M = 100 bp molecular DNA marker, N = RT negative control, 1–7 = samples).
(TIF)

S2 Fig Mucin producing cells in different groups of hamsters after 16 weeks of infection. (A) Mucin producing cells in H&E stained tissue sample of Cs+NDMA group hamsters (Original magnification ×200). (B) Bar diagram showing the number of mucin producing cells per tissue section in different hamster groups. The number of mucin producing cells in Cs+NDMA group is significantly higher compared to Cs or NDMA groups. *P* < 0.05 was considered as statistically significant. *P* < 0.001 indicated as a dagger.
(TIF)

S3 Fig Possible interactions among the genes/proteins in the development of *C*. *sinensis* induced CCA. The multifunctional gene *PSMD10* regulates CDK4 positively, but p53, *BAX* and RB negatively. Loss of cell cycle control due to the downregulation of RB and inactivation of apoptosis through reduced *p53*, *BAX* and *caspase 9* can promote the development of CCA.
(TIF)

S1 Table CCA differentiations (WDC, MDC and PDC) in different group of hamsters. (XLSX)

S2 Table CCA differentiations in Cs+NDMA group at different time points. (XLSX)

S3 Table Real-time PCR (SYBR Green I) based gene expressions in different group of hamsters. (XLSX)

S4 Table Densitometry (ImageJ of NIH) based protein expressions in different group of hamsters. (XLSX)

S5 Table Number of mucin producing cells per tissue sections in different group of hamsters. (XLSX)
