# Supplementary figures and images for: Involvement of PSMD10, CDK4, and Tumor Suppressors in Development of Intrahepatic Cholangiocarcinoma of Syrian Golden Hamsters Induced by Clonorchis sinensis and N-Nitrosodimethylamine
Source: PLoS Negl Trop Dis. 2015 Aug 27;9(8):e0004008. doi: 10.1371/journal.pntd.0004008 (PMC4551803; doi:10.1371/journal.pntd.0004008)

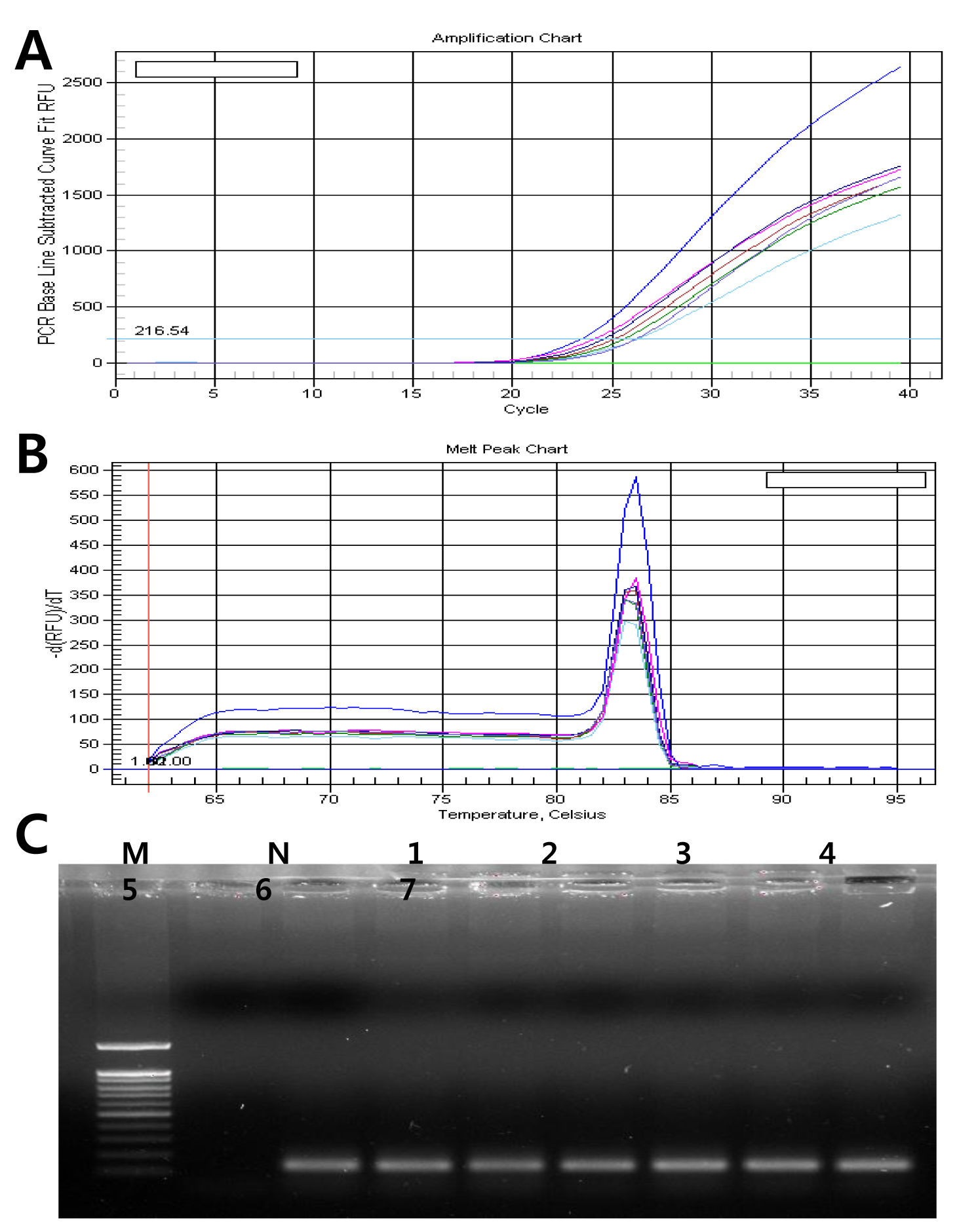

Supplement: S1 Fig — (A) Real-time amplification curve with negative RT product. (B) Melting peak chart of same amplified sample. (C) Agarose gel electrophoresis of same PCR product for the confirmation of single band (M = 100 bp molecular DNA marker, N = RT negative control, 1–7 = samples). (TIF) [file pntd.0004008.s001.tif]

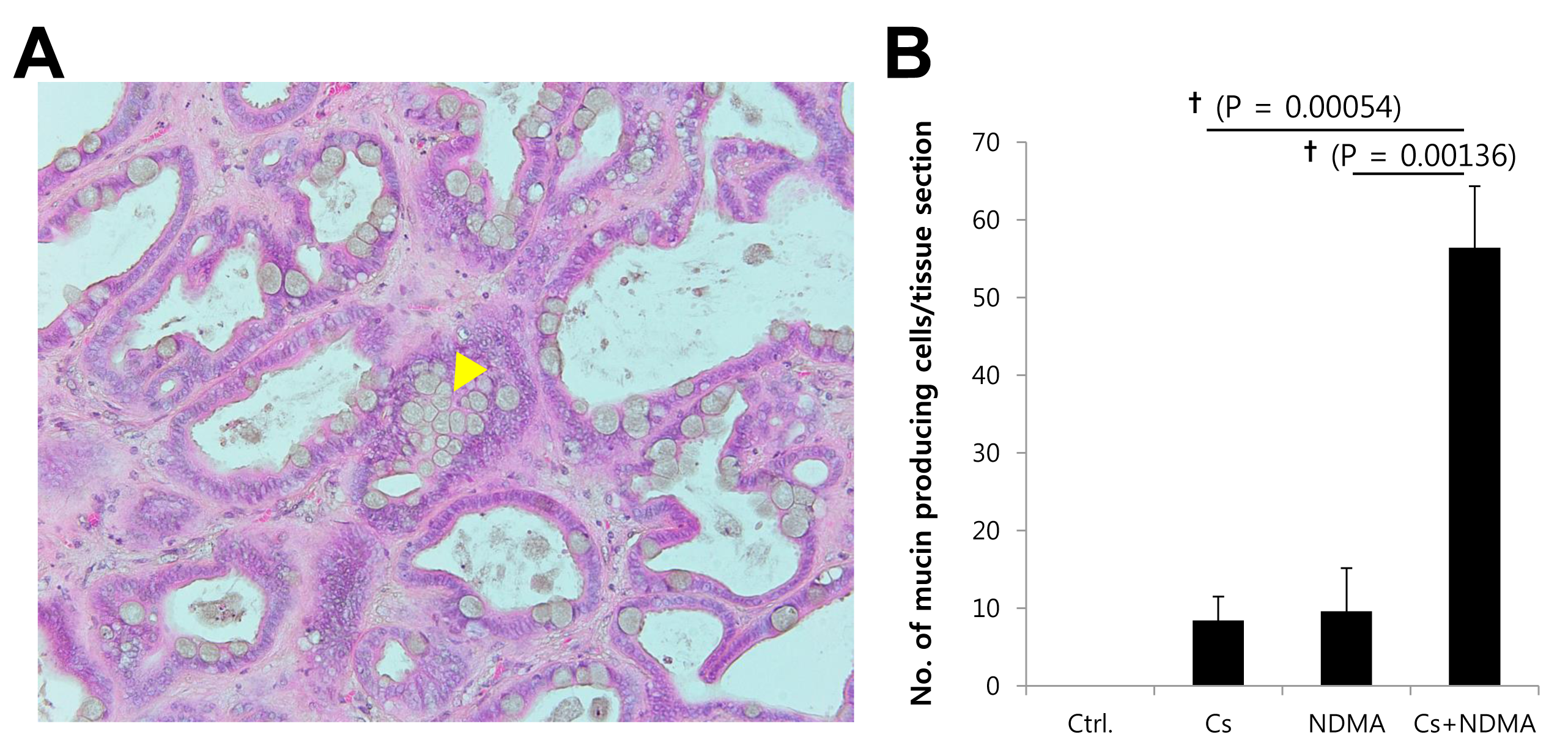

Supplement: S2 Fig — (A) Mucin producing cells in H&E stained tissue sample of Cs+NDMA group hamsters (Original magnification ×200). (B) Bar diagram showing the number of mucin producing cells per tissue section in different hamster groups. The number of mucin producing cells in Cs+NDMA group is significantly higher compared to Cs or NDMA groups. P < 0.05 was considered as statistically significant. P < 0.001 indicated as a dagger. (TIF) [file pntd.0004008.s002.tif]

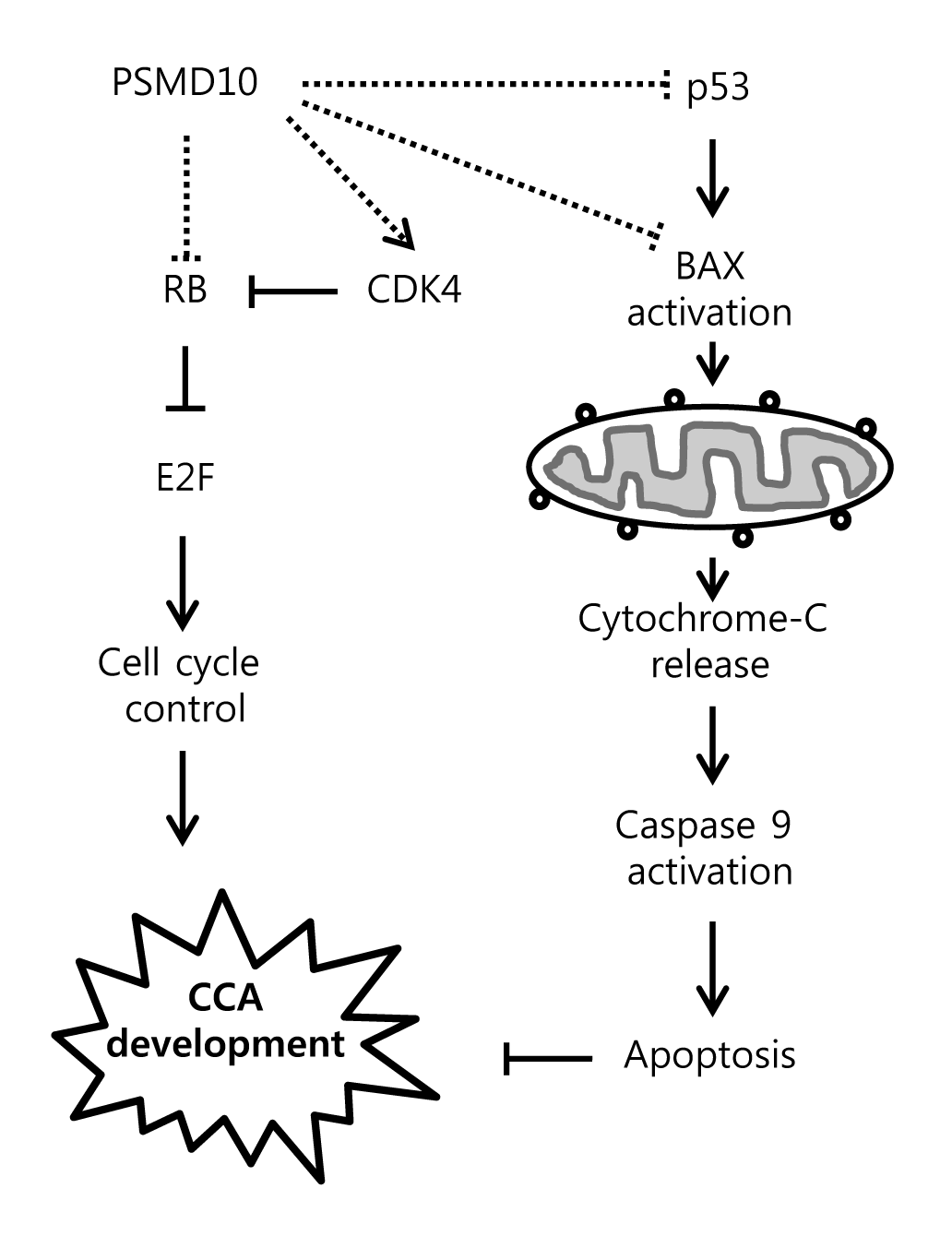

Supplement: S3 Fig — The multifunctional gene PSMD10 regulates CDK4 positively, but p53, BAX and RB negatively. Loss of cell cycle control due to the downregulation of RB and inactivation of apoptosis through reduced p53, BAX and caspase 9 can promote the development of CCA. (TIF) [file pntd.0004008.s003.tif]
